# Inhalation Exposure to PM_2.5_ Counteracts Hepatic Steatosis in Mice Fed High-fat Diet by Stimulating Hepatic Autophagy

**DOI:** 10.1038/s41598-017-16490-3

**Published:** 2017-11-24

**Authors:** Yining Qiu, Ze Zheng, Hyunbae Kim, Zhao Yang, Gary Zhang, Xiangyang Shi, Fei Sun, Changya Peng, Yuchuan Ding, Aixia Wang, Lung-Chi Chen, Sanjay Rajagopalan, Qinghua Sun, Kezhong Zhang

**Affiliations:** 1 Center for Molecular Medicine and Genetics, Wayne State University School of Medicine, Detroit, MI 48201 USA; 2 Department of Microbiology, Immunology, and Biochemistry, Wayne State University School of Medicine, Detroit, MI 48201 USA; 3 Department of Neurosurgery, Wayne State University School of Medicine, Detroit, MI 48201 USA; 4 Department of Physiology, Wayne State University School of Medicine, Detroit, MI 48201 USA; 5 Division of Cardiovascular Medicine, Davis Heart & Lung Research Institute, College of Medicine, Ohio State University, Columbus, OH 43210 USA; 6 Division of Environmental Health Sciences, College of Public Health, Ohio State University, Columbus, OH 43210 USA; 7 Case Cardiovascular Research Institute, Case Western Reserve University School of Medicine, 11100 Euclid Avenue, Cleveland, OH 44106 USA; 8 Department of Environmental Medicine, New York University, Tuxedo, NY 10987 USA; 9 Department of Pediatrics, Union Hospital, Tongji Medical College, Huazhong University of Science and Technology, Wuhan, 430022 PR China; 10 State Key Laboratory for Modification of Chemical Fibers and Polymer Materials, College of Chemistry, Chemical Engineering and Biotechnology, Donghua University, Shanghai, 201620 PR China

## Abstract

Air pollution is associated with the increased risk of metabolic syndrome. In this study, we performed inhalation exposure of mice fed normal chow or a high-fat diet to airborne fine particulate matters (PM_2.5_), and then investigated the complex effects and mechanisms of inhalation exposure to PM_2.5_ on hepatic steatosis, a precursor or manifestation of metabolic syndrome. Our studies demonstrated that inhalation exposure of mice fed normal chow to concentrated ambient PM_2.5_ repressed hepatic transcriptional regulators involved in fatty acid oxidation and lipolysis, and thus promoted hepatic steatosis. However, PM_2.5_ exposure relieved hepatic steatosis in high-fat diet-induced obese mice. Further investigation revealed that inhalation exposure to PM_2.5_ induced hepatic autophagy in mouse livers in a manner depending on the MyD88-mediated inflammatory pathway. The counteractive effect of PM_2.5_ exposure on high-fat diet-induced hepatic steatosis was mediated through PM_2.5_-induced hepatic autophagy. The findings from this study not only defined the effects and mechanisms of PM_2.5_ exposure in metabolic disorders, but also revealed the pleotrophic acts of an environmental stressor in a complex stress system relevant to public health.

## Introduction

Exposure to ambient fine particulate matter (aerodynamic diameter <2.5 μm, PM_2.5_) is known to be associated with cardiovascular and metabolic diseases, such as atherosclerosis, non-alcoholic fatty liver disease (NAFLD), and type-2 diabetes^1–4^. Depending on natural and anthropogenic emission sources, PM_2.5_ pollution is a complex mixture of chemical and biological elements, such as metals, salts, carbonaceous material, volatile organic compounds, polycyclic aromatic hydrocarbons, and endotoxins^5,6^. Airborne PM_2.5_ demonstrates an incremental capacity to penetrate to the most distal airway units and potentially the systemic circulation^7^. PM_2.5_, primarily derived from stationary and traffic-related combustion sources, has been demonstrated to trigger inflammatory stress responses that can remodel pathophysiology and thus promote metabolic disorders^2,4,8–11^. In this direction, we have revealed that inhalation exposure to PM_2.5_ has profound, direct effects on the liver, the major detoxification and metabolic organ^4,10,11^. PM_2.5_ exposure induces integrated oxidative stress, endoplasmic reticulum (ER) stress, and inflammatory responses in the liver, leading to a non-alcoholic steatohepatitis (NASH)-like phenotype, characterized by hepatic steatosis, inflammation, fibrosis, and insulin resistance, in animal models^4,10,11^.

Hepatic steatosis, the key feature of NAFLD, results from an imbalance between lipid synthesis, storage, oxidation, and/or secretion, and eventually causes hepatic injury^12,13^. Hepatic lipid metabolism is a highly coordinated process, in which many pathways are regulated by nuclear receptors, transcription factors, and cellular enzymes^14–18^. These factors integrate signals from various pathways and coordinate the activity of the metabolic machinery necessary for maintaining hepatic lipid homeostasis. Previously, we revealed that PM_2.5_ exposure alone causes hepatic steatosis and NASH by repressing metabolic regulators and up-regulating hepatic inflammatory signaling in animal models^4,11^. Since then, a critical question has been asked: does PM_2.5_ exposure interact with another risk factor, for examples, high-fat (HF) diet, to facilitate hepatic steatosis and the related metabolic symptoms?

In this study, we investigated whether PM_2.5_ exposure represents an additional “hit” that acts in synergy with the HF diet to promote NASH in HF-fed animals. In contract to our initial hypothesis that PM_2.5_ exposure interacts with the HF diet or obese conditions to exacerbate metabolic phenotypes, PM_2.5_ exposure counteracts hepatic steatosis and hyperlipidemia of obese mice under the HF diet through stimulating hepatic autophagy. Our study revealed the combined effects and mechanistic basis of two environmental stressors, airborne PM_2.5_ pollution and the HF diet, in modulating complex disease phenotypes. The findings from this study have important implications in the understanding, prevention, and treatment of the metabolic diseases associated air pollution.

## Results

### Inhalation exposure to PM_2.5_ relieves hepatic steatosis in animals under the HF diet

To determine the involvement of inhalation exposure to PM_2.5_ in metabolic complications, male C57BL/6 mice on normal chow or a HF diet (42% fat) were exposed to concentrated ambient PM_2.5_ or FA in exposure chambers of “OASIS” located at Columbus, USA, where most of the PM_2.5_ is attributed to long-range transport^2,4,11^. “OASIS” is a versatile aerosol concentration enrichment system (VACES) through which fine and ultrafine particles are concentrated and exposed to the animals in the chambers^19^. Prior to inhalation exposure to PM_2.5_, the animals of approximately 3 weeks of age were on normal chow or the HF diet for 10 weeks. PM_2.5_ inhalation exposure to the animals on normal chow or the HF diet was performed in a schedule of 6 hours a day, 5 days a week, which mimics the standard work schedule of professional highway automobile drivers or factory works, for a duration of 10 weeks. The ambient mean daily PM_2.5_ concentration at the study site was 15.8 μg/m^3^, whereas the mean concentration of PM_2.5_ in the exposure chamber was 111.0 μg/m^3^ ^8^. The elemental composition, as measured by energy-dispersive X-ray fluorescence (ED-XRF) analysis, as we previously reported^8,10^. The control mice in the experiment were exposed to an identical protocol with a high-efficiency particulate-air filter positioned in the inlet valve position to remove all of the PM_2.5_ in the filtered air (FA) stream.

**Figure 1:**
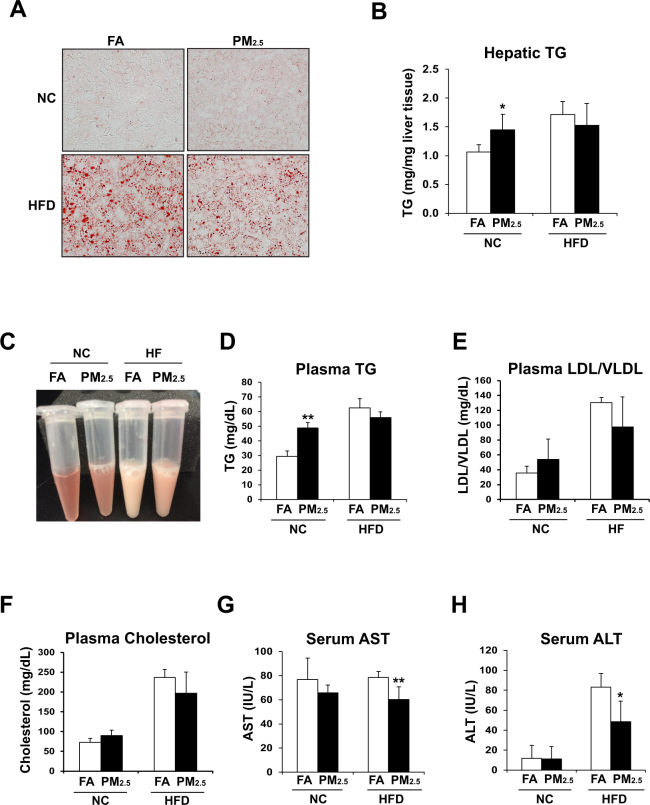
PM_2.5_ exposure induces hepatic steatosis in normal chow diet-fed mice but relieves hepatic steatosis in HF-fed mice. (**A**) Oil-red O staining of lipid droplets in the livers of the normal chow (NC)- or HF diet (HFD)- fed mice exposed to PM_2.5_ or FA for 10 weeks (magnification: 200x). (**B**) Levels of hepatic TG in the liver tissues of NC- or HFD- fed mice exposed to PM_2.5_ or FA for 10 weeks. N = 6 mice for PM_2.5_- or FA- exposed group under HFD; or 4 mice for PM_2.5_-or FA-exposed group under NC. Each bar denotes mean ± standard deviation (SD). *p < 0.05. **(C)** Photograph of blood-plasma samples from the representative NC- or HFD- fed mice after 10-week exposure to PM_2.5_ or FA. (**D–H**) Levels of blood TG (*D*), LDL/VLDL (*E*), total cholesterol (*F*), AST (*G*), and ALT (*H*) in NC- or HFD- fed mice exposed to PM_2.5_ or FA for 10 weeks. LDL/VLDL, low and very low density lipoproteins. Each bar denotes mean ± SD. *p < 0.05; **p < 0.01.

To evaluate the impact of inhalation exposure to concentrated PM_2.5_ on lipid homeostasis in the animals fed with normal chow or the HF diet, we examined lipid profiles with the liver tissues or blood samples of normal chow - or HF-fed mice exposed to PM_2.5_ or FA for 10 weeks. Oil-red O staining of liver tissue sections revealed that the animals under normal chow displayed significantly more lipid droplets accumulated in the liver after PM_2.5_ exposure, compared to the normal chow-fed animals under FA exposure (Fig. 1A). This supported the effect of PM_2.5_ exposure in inducing hepatic steatosis, as we previously demonstrated^4^. Surprisingly, the mice under the HF diet did not exhibit increased hepatic lipid accumulation after PM_2.5_ exposure, compared to the HF-fed animals under the FA exposure (Fig. 1A,B). Instead, PM_2.5_ exposure modestly reduced lipid accumulation in the livers of mice under the HF diet. The effects of PM_2.5_ exposure on increasing hepatic steatosis in the normal chow-fed animals but relieving hepatic steatosis in the HF-fed animals were confirmed by the enzymatic quantification assay of hepatic triglycerides (TG) (Fig. 1B).

**Figure 2:**
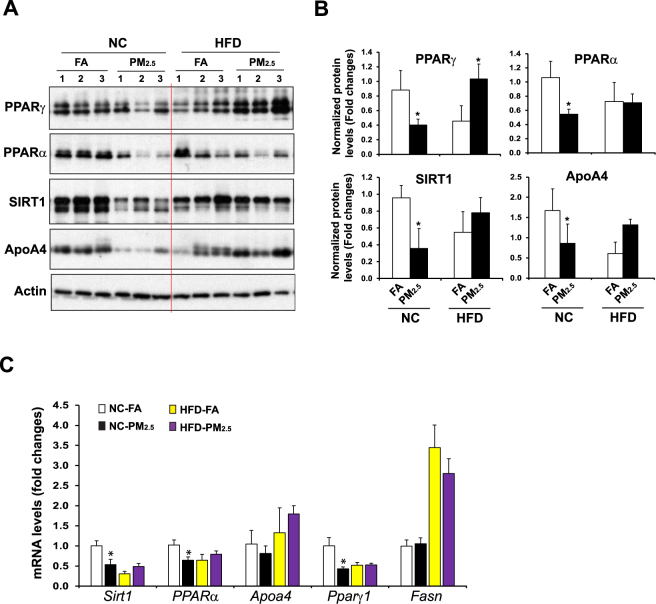
Expression of the key metabolic regulators in the livers of NC- or HFD- fed mice exposed to PM_2.5_ or FA. (**A**) Western blot analyses of levels of PPARγ, PPARα, SIRT1, and ApoA4 in the liver tissues from NC- or HFD- fed mice exposed to PM_2.5_ or FA for 10 weeks. Levels of β-actin were determined as loading controls. (**B**) Quantitative analyses of fold changes of PPARγ, PPARα, SIRT1, and ApoA4 protein levels, as determined by Western blot densitometry, in the livers of NC- or HFD- fed mice exposed to PM_2.5_ or FA. Each bar denotes mean ± SD. *p < 0.05. (**C**) Expression levels of *Sirt1*, *Pparα*, *Pparγ*, *Apoa4*, and *Fasn* mRNAs in the livers of NC- or HFD- fed mice exposed to PM_2.5_ or FA for 10 weeks. Expression levels of mRNAs were determined by qPCR. Fold changes of mRNA levels are shown by comparing to that of one of NC-fed mice under FA exposure. Each bar denotes mean ± SEM (n = 4 mice per group). *p < 0.05. *Fasn*, fatty acid synthase.

Next, we profiled blood lipids in the normal chow- or HF-fed mice exposed to PM_2.5_ or FA. Levels of blood TG, low/very low-density lipoproteins (LDL/VLDL), and total cholesterol were elevated in the mice under the normal chow diet exposed to PM_2.5_ for 10 weeks (Fig. 1C–F), confirming that PM_2.5_ exposure alone can promote hyperlipidemia in normal chow-fed animals^4^. However, upon PM_2.5_ exposure, the levels of circulating TG, LDL/VLDL, and total cholesterol in the animals under the HF diet were all repressed, compared to those of the FA-exposed animals under the HF diet (Fig. 1C–F). These results suggested that PM_2.5_ exposure can mitigate hyperlipidemia in obese animals induced by the HF diet. Furthermore, we examined serum levels of liver enzymes aspartate amino transferase (AST) and alanine aminotransferase (ALT), the indicators of liver damage^20^, in the PM_2.5_- or FA- exposed mice under the normal chow or HF diet. The levels of AST and ALT in the PM_2.5_-exposed mice under the HF diet were decreased, compared to those of the FA-exposed mice, under the normal chow diet (Fig. 1G–H). Additionally, the mice fed normal chow displayed modest body weight reduction after PM_2.5_, but the mice fed the HF diet showed no such reduction after PM_2.5_ exposure, compared to the FA-exposed mice fed the HF diet (S-Fig. 1). Consistent with our previous studies^4,10,11^, expression levels of the major pro-inflammatory genes encoding IL6 and TNFα in the livers of the mice under PM_2.5_ exposure were significantly increased, compared to those of the mice under FA exposure (S-Fig. 2). However, PM_2.5_-triggered increases in hepatic IL6 and TNFα expression were diminished in the liver of mice with deletion of Myeloid Differentiation Primary Response 88 (MyD88), the key mediator of the toll-like receptor (TLR) 2- and TLR 4-mediated inflammatory pathways^21^, suggesting that MyD88 plays a key regulatory role in PM_2.5_-stimulted hepatic pro-inflammation. Together, these results were consistent with the alleviated hepatic steatosis and hyperlipidemia in the HF-fed animals upon PM_2.5_ exposure, suggesting the effects of PM_2.5_ exposure on counteracting hepatic steatosis and hyperlipidemia as well as relieving liver injuries in the HF-fed animals.

### PM_2.5_-caused repression on the key metabolic regulators in the liver is attenuated under the HF-feeding condition

Hepatic transcription factors or nuclear receptors play major roles in maintaining lipid homeostasis under pathophysiological stress conditions^14,22^. To determine the molecular basis underlying the effects of PM_2.5_ exposure on modulating lipid homeostasis under the normal chow or HF diet, we examined expression of the major metabolic and inflammatory regulators that are functionally involved in FA oxidation, lipolysis, and anti-inflammation in the livers of mice under normal chow or the HF diet. Under the normal chow diet, inhalation exposure to PM_2.5_ significantly repressed expression of PPARγ, a major anti-inflammatory regulator, in mouse livers, as revealed by Western blot and qPCR analyses (Fig. 2A–C). Further, expression of PPARα, SIRT1 and ApoA4, the major hepatic regulators of FA oxidation and lipolysis in the liver, was also repressed by PM_2.5_ exposure. These data are consistent with the effect of PM_2.5_ exposure on promoting hepatic steatosis. In contrast, under the HF feeding condition, expression levels of PPARγ, PPARα, SIRT1, and ApoA4 in the livers of the PM_2.5_-exposed mice were higher than those in the FA-exposed mice (Fig. 2A–C). The up-regulation of the major hepatic metabolic regulators by PM_2.5_ exposure in HF-fed animals confirmed the effect of inhalation exposure to PM_2.5_ on relieving hepatic steatosis and hyperlipidemia caused by HF feeding.

### Inhalation exposure to PM_2.5_ induces hepatic autophagy

**Figure 3:**
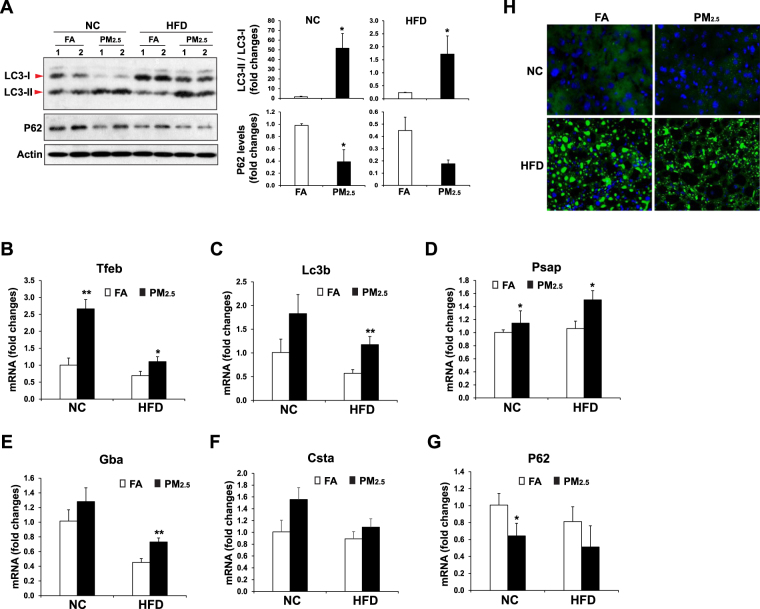
Inhalation exposure to PM_2.5_ induces autophagy in the livers of mice under NC or HFD. (**A**) Western blot analyses of levels of LC3 and P62 in the liver tissues from NC- or HFD- fed mice exposed to PM_2.5_ or FA for 10 weeks. Levels of β-actin were determined as loading controls. The graphs beside the images show the quantitative analyses of fold changes of ratios of LC3-II vs LC3-I and P62 protein levels, as determined by Western blot densitometry, in the livers of NC- or HFD- fed mice exposed to PM_2.5_ or FA. The protein level was normalized to that of β-actin before fold change calculations. Each bar denotes mean ± SD. *p < 0.05. (**B**–**G**) Expression levels of *Tfeb*, *Lc3b*, *Psap* (Prosaposin), *Gba* (Glucosidase, beta, acid), *Csta* (Cystatin A), and *P62* mRNAs in the livers of NC- or HFD- fed mice exposed to PM_2.5_ or FA for 10 weeks. Expression levels of mRNAs were determined by qPCR. Fold changes of mRNA levels are shown by comparing to that of one of NC-fed mice under FA exposure. Each bar denotes mean ± SEM (n = 4 mice per group). *p < 0.05, **p < 0.01. (**H**) BODIPY staining of lipid droplets in livers of NC- or HFD- fed mice exposed to PM_2.5_ or FA for 10 weeks (magnification: 200×).

We sought to understand the regulatory mechanism by which PM_2.5_ exposure counteracts hepatic steatosis in the animals under the HF diet. Among many stress-induced regulatory pathways we investigated, autophagy is a lysosomal degradative pathway that regulates cellular functions relevant to NAFLD^23,24^. It has been demonstrated that autophagy regulates lipid metabolism by eliminating TG and by preventing development of steatosis^25^. While autophagy participates in the basal turnover of lipids by engulfing and degrading lipid droplets under physiological conditions, excessive lipids, induced by HF feeding, inhibits hepatic autophagic turnover^25^. Autophagy can be measured by induction and conversion of microtubule-associated protein 1 A/1B-light chain 3 (LC3), as levels of conversion of LC3-I to LC3-II provides a reliable indicator of autophagic activity^26^. To test whether inhalation exposure to PM_2.5_ induce autophagy in the liver, we examined induction and conversion of LC3 in the livers of normal chow- or HF- fed mice under PM_2.5_- or FA- exposure for 10 weeks. In response to PM_2.5_ exposure, the levels of LC3-II proteins were significantly increased in the livers of mice under either normal chow or the HF diet (Fig. 3A), indicating a strong effect of PM_2.5_ exposure on triggering hepatic autophagy activity. It is important to note that the levels of LC3-I to LC3-II conversion were reduced in the livers of the HF-fed animals, compare to those of the normal chow-fed mice (Fig. 3A), consistent with the notion that HF feeding, obesity, or hepatic steatosis reduced hepatic autophagy activity^27–29^. However, after 10-week inhalation exposure to PM_2.5_, but not FA, the levels of LC3-II protein in the livers of the HF-fed mice were significantly up-regulated, to the levels comparable to that in normal chow-fed mice after PM_2.5_ exposure (Fig. 3A), suggesting a significant role of PM_2.5_ exposure in counteracting the suppressive effect of HF feeding or hepatic steatosis on hepatic autophagy. The PM_2.5_-induced hepatic autophagy activities were confirmed by the decreased levels of P62 protein, a receptor for cargo destined to be degraded by autophagy, in the livers of PM_2.5_-exposed mice under normal chow or the HF diet (Fig. 3A).

Further, we examined expression of the genes encoding the key regulators, components or targets of the autophagic pathway in the livers of FA- or PM_2.5_- exposed mice under normal chow or the HF diet. Expression levels of the gene encoding Transcription Factor EB (TFEB), a master regulator of lysosomal biogenesis^30^, were significantly increased in the livers of the mice exposed to PM_2.5_, compared to those of mice exposed to FA (Fig. 3B). Similarly, expression levels of the genes encoding the key components or targets of autophagy, including LC3b, PSAP, GBA, CSTA, and P62, were also significantly increased in the livers of PM_2.5_-exposed mice (Fig. 3C–G), thus confirming the hepatic autophagy activity triggered by PM_2.5_ exposure. Additionally, to verify the specific effect of PM_2.5_ on triggering autophagy in hepatocytes, human hepatocyte cell line Huh7 was cultured in the conditioned medium from the macrophage cell line RAW264.7 exposed to PM_2.5_ or vehicle for 28 h. Upon PM_2.5_ exposure, both the level of LC3-II protein and the ratio of LC3-II *vs* LC3-I were increased in Huh7 cells (S-Fig. 3). Treatment of the specific autophagy inhibitor 3-Methyladenine (3-MA) suppressed PM_2.5_-induced hepatic autophagy, as the level of cleaved LC3-II was reduced in the Huh7 cells challenged with PM_2.5_ in combination with 3-MA, compared to that in the Huh7 cells challenged with PM_2.5_ alone (S-Fig. 3). This result confirmed the specific effect of PM_2.5_ exposure on triggering hepatic autophagy.

The ability of autophagy to degrade lipid droplets in liver hepatocytes has been specifically termed lipophagy^25^. To determine whether PM_2.5_-triggered hepatic autophagy can down-regulate lipid droplets in HF-fed mouse livers through lipophagy, we examined lipid droplets in the livers of PM_2.5_- or FA-exposed mice under normal chow or the HF diet. BODIPY staining of hepatic lipid droplets showed that the mass and size of lipid droplets in the livers of PM_2.5_-exposed mice were reduced, compared to those of FA-exposed mice, under the HF diet (Fig. 3H). This is consistent with the reduced hepatic TG levels as shown in Fig. 1A. Together, these data validated the effect of PM_2.5_ exposure in relieving hepatic steatosis through lipophagy.

### PM_2.5_-triggered hepatic autophagy and its counteractive effect on hepatic steatosis rely on MyD88

**Figure 4:**
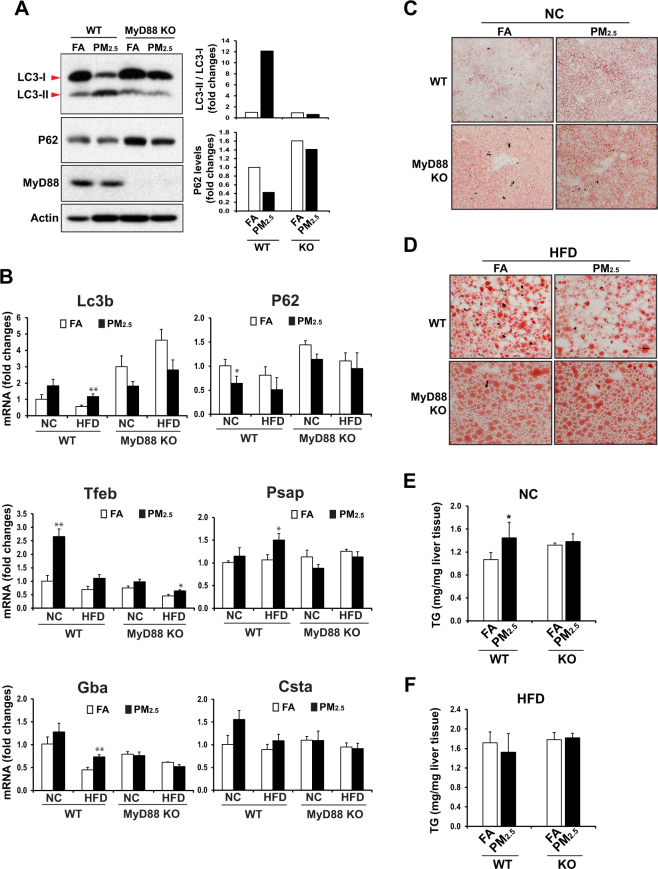
MyD88 is required for PM_2.5_-triggered hepatic autophagy and its counteractive effect on hepatic steatosis. (**A**) Western blot analyses of levels of LC3, P62, MyD88, and β-actin in the liver tissues of NC-fed WT or MyD88 KO mice exposed to PM_2.5_ or FA for 10 weeks. Liver protein lysates were prepared from pooled liver tissues of each group of mice (n = 4 mice per group). The graphs beside the images show the quantitative analyses of fold changes of ratios of LC3-II vs LC3-I and P62 protein levels, as determined by Western blot densitometry, in the livers of NC- or HFD- fed mice exposed to PM_2.5_ or FA. The protein level was normalized to that of β-actin before fold change calculations. **(B)** Expression levels of *Lc3b*, *P62*, *Tfeb*, *Psap*, *Gba*, and *Csta* mRNAs in the livers of the PM_2.5_- or FA- exposed WT and MyD88 KO mice under NC or HFD. Expression levels of mRNAs were determined by qPCR. Fold changes of mRNA levels are shown by comparing to that of one of NC-fed mice under FA exposure. Each bar denotes mean ± SEM (n = 4 mice per group). *p < 0.05, **p < 0.01. **(C–D)** Oil-red O staining of lipid droplets in the livers of NC- or HFD- fed WT and MyD88 KO mice exposed to PM_2.5_ or FA for 10 weeks (magnification: 200×). (**E–F)** Levels of hepatic TG in the liver tissues of NC- or HFD- fed WT and MyD88 KO mice exposed to PM_2.5_ or FA for 10 weeks. N = 4–6 mice per group. Each bar denotes mean ± SD. *p < 0.05.

We gained further insight into the regulatory mechanism by which PM_2.5_ exposure induces hepatic autophagy. Our previous works demonstrated that inhalation exposure to PM_2.5_ strongly induces TLR2- and TLR4- mediated inflammatory responses in the circulating monocytes and in the liver^4,11,31^. MyD88 is a critical adaptor protein used by almost all TLRs to mediate the downstream TLR signaling pathways^21^. To elucidate the involvement of TLR signaling pathway in PM_2.5_-triggered hepatic autophagy, MyD88 knockout (KO) and wild-type (WT) control mice were subjected to inhalation exposure to PM_2.5_ or FA for 10 weeks. We examined expression and conversion of LC3 protein in the liver of PM_2.5_- or FA- exposed MyD88 KO and WT control mice. While PM_2.5_ exposure increased the levels of LC3-II protein in the livers of WT mice, it failed to increase the levels of LC3-II in the livers of MyD88 KO mice (Fig. 4A). Indeed, the levels of LC3-II protein in the livers of PM_2.5_-exposed MyD88 KO mice were comparable to those in the FA-exposed WT or MyD88 KO mice, suggesting that MyD88 is required for the induction of hepatic autophagy triggered by PM_2.5_ exposure. The inability of PM_2.5_ exposure to induce hepatic autophagy in MyD88 KO mice was confirmed by the increased levels of P62 proteins in the livers of MyD88 KO mice under PM_2.5_ or FA exposure (Fig. 4A). Furthermore, we examined expression of the genes encoding the key regulators or components of the autophagic pathway, including LC3b, P62, TFEB, PSAP, GBA, and CSTA, in the livers of WT and MyD88 KO mice under the normal chow or HF diet. Expression levels of the *Lc3b*, *Tfeb*, *Psap*, *Gba*, and *Gsta* genes in the livers of normal chow- or HF-fed MyD88 KO mice were reduced, compared to those in WT control mice, especially after PM_2.5_ exposure (Fig. 4B). Moreover, the expression levels of the *P62* gene in the livers of MyD88 KO mice were increased, compared to that of WT mice, implicating decreased hepatic autophagy in the absence of MyD88. Taken together, these data suggested that hepatic autophagy activation triggered by PM_2.5_ exposure relies on MyD88.

As MyD88 is required for PM_2.5_-triggered hepatic autophagy, we evaluated whether the counteractive effect of PM_2.5_ on hepatic steatosis relies on MyD88. To address this question, we first evaluated lipid droplet accumulation in the livers of MyD88 KO and WT control mice under normal chow or the HF diet after PM_2.5_ or FA exposure. Under the normal chow diet, FA-exposed WT mice did not display any hepatic steatosis, while PM_2.5_ exposure increased lipid droplet accumulation in the liver, leading to discernable hepatic steatosis in WT mice (Fig. 4C). As we demonstrated previously^4^ and as shown in Fig. 2, PM_2.5_-triggered hepatic lipid accumulation was attributed to, at least in part, the repression of the key metabolic regulators in FA oxidation and lipolysis. Apparently, repression of FA oxidation and lipolysis under PM_2.5_ exposure overrode the anti-hepatic steatosis effect of PM_2.5_-triggered hepatic autophagy under normal chow, and thereby led to hepatic steatosis in WT animals. However, oil-red O staining of hepatic lipids indicated that MyD88 KO mice exhibited hepatic steatosis even under FA exposure, although PM_2.5_ exposure further exacerbated hepatic steatosis in MyD88 KO mice (Fig. 4C). These results were confirmed by the quantitative assay of hepatic TG in the MyD88 KO and WT mice exposed to FA or PM_2.5_ (Fig. 4E). Together, these data support the anti-steatosis effect of PM_2.5_-triggered hepatic autophagy and the requirement of MyD88 for PM_2.5_-triggered hepatic autophagy. Moreover, we evaluated the impact of MyD88 deficiency in hepatic steatosis in PM_2.5_-exposed, HF-fed animals. While PM_2.5_ exposure relieved hepatic steatosis in HF-fed WT mice, the anti-hepatic steatosis effect of PM_2.5_ exposure was diminished in the HF-fed MyD88 KO mice (Fig. 4D,F). Indeed, hepatic lipid accumulation in PM_2.5_-exposed MyD88 KO mice was slightly increased, compared to that in FA-exposed MyD88 KO mice, under the HF diet. These results further confirmed that PM_2.5_-triggered hepatic autophagy and its counteractive effect on hepatic steatosis in HF-fed animals rely on MyD88.

## Discussion

**Figure 5:**
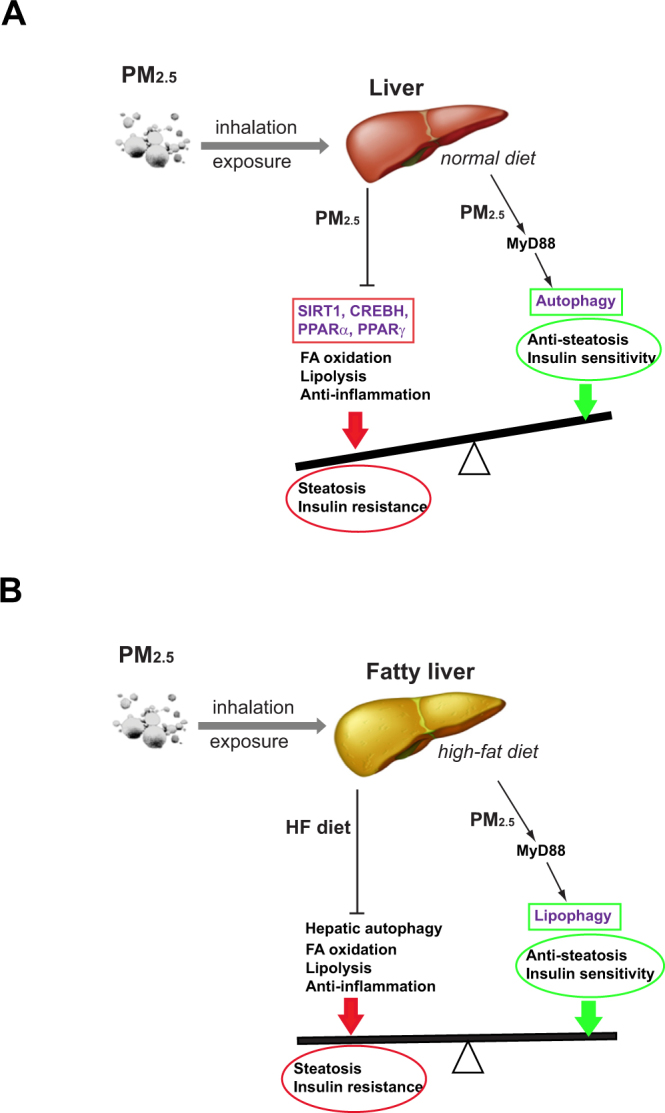
The working models for the effect of PM_2.5_ exposure in promoting or relieving hepatic steatosis in mice under NC or HFD. (**A**) The effects and pathways by which inhalation exposure to PM_2.5_ represses lipid metabolic and anti-inflammatory pathways, and induces hepatic autophagy in the livers of mice under NC. Because the repression of hepatic lipid metabolic pathways overweigh the anti-steatosis effect of hepatic autophagy triggered by PM_2.5_ exposure, the NC-fed mice exhibited increased hepatic steatosis upon PM_2.5_ exposure. (**B**) The effects and pathways by which PM_2.5_ exposure and HF feeding repress or promote hepatic autophagy, lipid metabolic and anti-inflammatory pathways in the livers of mice under HFD. Because HF feeding represses hepatic autophagy or lipophagy, a major cause of HF-induced hepatic steatosis, PM_2.5_-triggered hepatic autophagy exerts a discernable effect on mitigating hepatic steatosis in the HF-fed animals. The illustrations were generated by using Adobe Illustrator software.

In this study, we used a “real-world” PM_2.5_ exposure system to perform ambient inhalation exposure of experimental animal models to environmentally relevant PM_2.5_. Through this system, we delineated the effects and molecular mechanisms for PM_2.5_ alone or in combination with another risk factor, specifically the HF or “Western-style” diet, in hepatic steatosis. Our study revealed an unexpected “beneficial” effect of inhalation exposure to PM_2.5_ on counteracting hepatic steatosis of HF-fed or obese individuals, and linked hepatic autophagic flux with PM_2.5_ exposure. The major findings from this study include (Fig. 5): 1) while inhalation exposure to PM_2.5_ induces hepatic steatosis and hypertriglyceridemia in normal chow-fed mice, PM_2.5_ exposure attenuates hepatic steatosis and hyperlipidemia in HF-fed mice; 2) PM_2.5_ exposure mitigates liver injuries of HF-fed mice; 3) PM_2.5_ exposure-caused repression of the key metabolic regulators PPARα, PPARγ and SIRT1 in the liver are attenuated under the HF-feeding condition; 4) PM_2.5_ exposure induces hepatic autophagy; 5) PM_2.5_-triggered anti-hepatic steatosis effect in HF-fed animals is mediated through hepatic autophagy; and 5) PM_2.5_-triggered hepatic autophagy and its counteractive effect on hepatic steatosis rely on MyD88, the key mediator of TLR-mediated inflammatory signaling. These findings not only have important implications in the understanding of the complex effects of airborne PM_2.5_ pollution on metabolic disease, but also provide novel insights into the mechanistic basis by which multiple stressors act to modulate pathophysiology in a complex system relevant to public health.

Previously, we revealed that inhalation exposure to PM_2.5_ induce a NASH-like phenotype, characterized by hepatic steatosis, inflammation, fibrosis as well as hepatic glycogen depletion, in mice under the normal chow diet^4,11^. Because HF diet is a common health risk factor associated with the development of metabolic disorders, we asked whether airborne PM_2.5_ pollution can synergize with HF diet to facilitate metabolic symptoms. Indeed, previous studies showed that inhalation exposure to PM_2.5_ promoted atherosclerosis in Apolipoprotein E knockout (ApoE^−/−^) under a HF diet^1^. Therefore, it was tempted to hypothesize that PM_2.5_ exposure may interact with the HF diet to exacerbate hepatic steatosis and the associated metabolic syndrome. Surprisingly, our study with the HF-fed animals revealed an unexpected “beneficial” effect of PM_2.5_ exposure on counteracting hepatic steatosis induced by the HF diet. While inhalation exposure to high levels of PM_2.5_ leads to non-alcoholic liver steatosis in normal chow-fed mice, PM_2.5_ exposure relieves hepatic lipid accumulation in mice under the HF diet, which is achieved by hepatic-specific autophagy (lipophagy) triggered by PM_2.5_. In addition to hepatic steatosis, the levels of liver injuries as well as circulating TG and cholesterol were also reduced in the HF-fed, PM_2.5_-exposed animals, compared to those of the HF-fed, FA-exposed animals (Fig. 1). These results confirmed PM_2.5_-triggered anti-hepatic steatosis, anti-hyperlipidemia, and anti-liver injury effects in HF-fed or obese animal models. This scenario shifts our understanding of the “two hits” hypothesis that two stressors or insults act together to facilitate NAFLD pathogenesis^32^. In our model system, the second “hit”, PM_2.5_ exposure, counteracts the first “hit”, the HF diet, in driving hepatic steatosis. This is in line with the challenge of using two-hit hypothesis to explain complex diseases including NAFLD^33^. It was suggested that multiple insults act together on genetically predisposed subjects to induce NAFLD. Such hits include insulin resistance, nutritional factors, gut microbiota and genetic and epigenetic factors. Our work provides a new dimension in regard to the effects of multiple stressors or hits on driving NAFLD pathogenesis. The environmental stressor, PM_2.5_ exposure, mitigates the effect of the nutritional factor, the high-fat diet, in promoting hepatic steatosis. This finding represents an important new contribution to the understanding of the complex effects of multiple stressors and the stress mechanism of NAFLD.

The anti-steatosis effect resulted from inhalation exposure to PM_2.5_ has been explained by the findings from this study (Fig. 5). Our study revealed that PM_2.5_ exposure is a strong trigger of hepatic autophagy program in a manner depending on the inflammatory pathway mediated through MyD88. Autophagy has important protective metabolic functions, including maintenance of insulin sensitivity and degradation of intracellular lipids, under metabolic conditions, such as obesity or HF feeding^25,27^. HF diet-induced obesity leads to decreased autophagy in the liver^25,27^, and loss of autophagic function is involved in the pathogenesis of NAFLD^28,29^. Our studies indicate that the anti-hepatic steatosis effect of PM_2.5_ exposure is consistent with the metabolic functions of hepatic autophagy. Under normal chow, PM_2.5_ exposure triggers hepatic autophagy, but meanwhile represses FA oxidation and lipolysis (Fig. 5A). Because the repression of FA oxidation and lipolysis overrode the effect of hepatic autophagy, the normal chow-fed animals exhibited discernable hepatic steatosis under PM_2.5_ exposure. Under the HF diet, levels of hepatic autophagy were reduced - a major cause of HF diet-induced hepatic steatosis, obesity, or type-2 diabetes^25,27,28^. Under the condition of HF diet-induced hepatic steatosis, PM_2.5_-triggered hepatic autophagy (lipophagy) counteracts the effect of HF feeding on repressing autophagy and potentially other metabolic pathways. In another word, the fat-relieving effect of PM_2.5_-induced lipophagy overweighed the repressive effect of lipid mobilization caused by exposure to PM_2.5_, and therefore, PM_2.5_ exposure exhibited discernable effects on improving hepatic steatosis, liver injuries, and hyperlipidemia in the HF-fed animals (Fig. 5B).

Our study also raised an interesting question: whether the individuals with obesity or NAFLD resulted from HF or “Western” diet can gain health benefits by living in the areas under high-levels of ambient PM_2.5_? It is immature to make such an assumption, because: 1) we do not know whether the effect of PM_2.5_ exposure on reliving hepatic steatosis is associated with the length of exposure time. The results from this study were based on the mice under 10-week PM_2.5_ exposure. Does longer- or shorter- term of PM_2.5_ exposure exert the same effect on boosting hepatic autophagy and reliving hepatic steatosis in HF-fed animals? In addition to the relatively short duration of PM_2.5_ exposure, animal studies can only provide a snap shot of a continuing interplay between injury and repair. The net outcome could shift to the other directions if these endpoints were taken at a different time point. PM_2.5_ exposure may trigger a shift in metabolic endpoints indicating that prolonged and continuous exposure to PM_2.5_ pollution could ultimately lead to adverse health outcomes in HF-fed animals. 2) While PM_2.5_ exposure has the beneficial effect on improving hepatic steatosis and hyperlipidemia for the individuals under the HF diet, PM_2.5_-triggered systemic responses may have detrimental effects on other organs or tracks, such as adipose tissue, cardiovascular system, and neuronal system. Nevertheless, our finding is significant since it increases our understanding of the complex effects and mechanistic basis of stress responses in complex systems and of the complexity of pollution-diet interactions. Multiple stressors or hits may not act in synergy to exacerbate disease pathogenesis. It is possible that multiple stressors counteract with each other and therefore complicate the pathophysiological impact of “beneficial” *vs* “detrimental” stressors or interventions. This knowledge has important implications in the understanding of modern human complex diseases and pharmaceutical interventions towards the cure of these diseases.

## Materials and Methods

### Exposure of animals to ambient PM_2.5_

Mice were exposed to concentrated ambient PM_2.5_ or filtered air (FA) in a mobile trailer “Ohio’s Air Pollution Exposure System for the Interrogation of Systemic Effects (OASIS)” in Columbus, OH, where most of the PM_2.5_component is attributed to long-range transport^2,4,11^. The concentrated PM_2.5_ was generated using a versatile aerosol concentration enrichment system (VACES) as described previously^19^. C57BL/6 male mice of six-week-old were purchased from the Jackson Laboratories (Bar Harbor, ME), and were equilibrated for 2 weeks prior to experimental enrollment. The mice were housed in cages with normal chow diet or a high-fat diet (Teklad TD 88137, 42% calories from fat) in an Association for Assessment and Accreditation of Laboratory Animal Care-accredited animal housing facility. Mice were exposed to concentrated PM_2.5_ at nominal 10 × ambient concentrations 6 hours per day, 5 days per week for a total of 10 weeks, as detailed previously^10,34^. The control (FA) mice in the experiment were exposed to an identical protocol with the exception of a high-efficiency particulate-air (HEPA) filter positioned in the inlet valve position to remove all of the PM_2.5_ in the filtered air stream. All the animal experiments were approved by the Ohio State University and the Wayne State University IACUC committee and carried out under the institutional guidelines for ethical animal use.

### Oil-red O staining

Frozen liver tissue sections were stained with Oil-red O for lipid contents according to standard protocol^35^. Briefly, frozen liver tissue sections of 8 µm were air-dried, and then fixed in formalin. The fixed sections were rinsed in 60% isopropanol followed by staining with freshly prepared Oil-red O solution for 15 minutes. After Oil-red O staining, liver sections were rinsed in 60% isopropanol followed by washing with water before subjected to microscope analysis.

### BODIPY staining of lipid droplets

the OCT-embedded, frozen mouse liver tissue sections were fixed with 3% formaldehyde for 15 min and stained with BODIPY (boron dipyrromethene) 493/503 (1 μg/ml) for 15 min at room temperature. The stained liver tissue sections were mounted with Prolong gold antifade reagent containing DAPI (4′,6-diamidino-2-phenylindole).

### Measurement of mouse lipid contents

To determine hepatic TG levels, approximately 100 mg mouse liver tissue was homogenized in PBS followed by centrifugation. The supernatant was mixed with 10% Triton-100 in PBS for TG measurement using a commercial kit (BioAssay Systems, Hayward, CA). Levels of plasma TG, total cholesterol, high-density lipoprotein (HDL), and low and very low density lipoprotein (LDL/VLDL) in the mice were determined enzymatically using commercial kits (Roche Diagnostics Corporation).

### Western blot analyses

Total mouse liver protein lysates were prepared using NP-40 lysis buffer supplemented with protease inhibitors (EDTA-free Complete Mini, Roche). Denatured proteins were separated by SDS-PAGE on 10% Tris-glycine polyacrylamide gels and transferred to a 0.45-mm PVDF membrane (GE Healthcare). Membrane-bound antibodies were detected by an enhanced chemiluminescence detection reagent (GE Healthcare). Quantification of Western blot signal intensities were determined by Quantity One 4.6.7 (Bio-Rad Life Science, CA).

### Quantitative real-time RT-PCR (qPCR) analysis

For real-time PCR analysis, the reaction mixture containing cDNA template, primers, and SYBR Green PCR Master Mix (Applied Biosystems) was run in a 7500 Fast Real-time PCR System (Applied Biosystems, Carlsbad, CA). The sequences of real-time PCR primers used in this study are shown in Supplemental Table 1. Fold changes of mRNA levels were determined after normalization to internal control β-actin RNA levels.

### Statistics

All *in vitro* experiments were repeated with biological triplicates at least three times independently. The data have been analyzed and compared by an unpaired, 2-tailed Student’s *t* test. Statistical tests with *p* < 0.05 were considered significant.

## Electronic supplementary material

Supplemental information
