# Supplementary material for: Inhalation Exposure to PM2.5 Counteracts Hepatic Steatosis in Mice Fed High-fat Diet by Stimulating Hepatic Autophagy
Source: Sci Rep. 2017 Nov 24;7:16286. doi: 10.1038/s41598-017-16490-3 (PMC5701159; doi:10.1038/s41598-017-16490-3)
Supplement: Supplementary file 1 — Supplemental information [file 41598_2017_16490_MOESM1_ESM.pdf]

Inhalation Exposure to $PM_{2.5}$ Counteracts Hepatic Steatosis in Mice Fed High-fat Diet by Stimulating Hepatic Autophagy

Yining Qiu[1,9], Ze Zheng[1], Hyunbae Kim[1], Yang Zhao[1], Gary Zhang[1], Xiangyang Shi[10], Fei Sun[4], Changya Peng[3], Yuchuan Ding[3], Aixia Wang[5], Lung Chi Chen[8], Sanjay Rajagopalan[7], Qinghua Sun[5,6], and Kezhong Zhang[1, 2*]

From [1] Center for Molecular Medicine and Genetics, [2] Department of Microbiology, Immunology, and Biochemistry, [3] Department of Neurosurgery, [4] Department of Physiology, Wayne State University School of Medicine, Detroit, MI 48201, USA;

[5] Division of Cardiovascular Medicine, Davis Heart & Lung Research Institute, College of Medicine, [6] Division of Environmental Health Sciences, College of Public Health, Ohio State University, Columbus, OH 43210, USA;

[7] Case Cardiovascular Research Institute, Case Western Reserve University School of Medicine, 11100 Euclid Avenue, Cleveland, OH, 44106, USA

[8] Department of Environmental Medicine, New York University, Tuxedo, NY 10987, USA.

[9] Department of pediatrics, Union Hospital, Tongji Medical College, Huazhong University of Science and Technology, Wuhan 430022, PR China

[10] State Key Laboratory for Modification of Chemical Fibers and Polymer Materials, College of Chemistry, Chemical Engineering and Biotechnology, Donghua University, Shanghai 201620, PR China

Running title: $PM_{2.5}$ exposure counteracts hepatic steatosis in obesity

*Corresponding Athours:
Kezhong Zhang, Ph.D., 540 E. Canfield Avenue, Detroit, MI 48201
Tel: 313-577-2669; FAX: 313-577-5218; Email: kzhang@med.wayne.edu

Supplemental figure legends

S-figure 1. Body weights of the normal chow- or HF- fed mice exposed to $PM_{2.5}$ or FA for 10 weeks. N = 6 mice for $PM_{2.5}$- or FA- exposed group under the HF diet; or 4 mice for $PM_{2.5}$- or FA-exposed group under the normal chow diet. Each bar denotes mean ± SEM.

S-figure 2. Expression levels of the *IL6* and *TNFα* mRNAs in the livers of the $PM_{2.5}$- or FA-exposed WT and MyD88 KO mice. Expression levels of mRNAs were determined by qPCR. Fold changes of mRNA levels are shown by comparing to that of one of NC-fed mice under FA exposure. Each bar denotes mean ± SEM (n = 4 mice per group). * $p < 0.05$.

S-figure 3. Western blot analyses of levels of LC3 protein in Huh7 cells cultured in the conditioned medium from RAW264.7 cells exposed to $PM_{2.5}$ (5μg/mL), $PM_{2.5}$ plus 3-MA (2mM), or vehicle control PBS (Ctl) for 28 h. Huh7 cells were incubated with the conditioned medium for 36 h before subjected to Western blot analysis. Levels of GAPDH were determined as loading controls. The graph beside the images showes the quantitative analyses of fold changes of ratios of LC3-II vs LC3-I protein levels, as determined by Western blot densitometry, in Huh7 cells exposed to $PM_{2.5}$, $PM_{2.5}$ plus 3-MA, or control medium. The protein level was normalized to that of GAPDH before fold change calculations.

S-table 1. Primer sequence information for the real-time PCR analysis in this study.

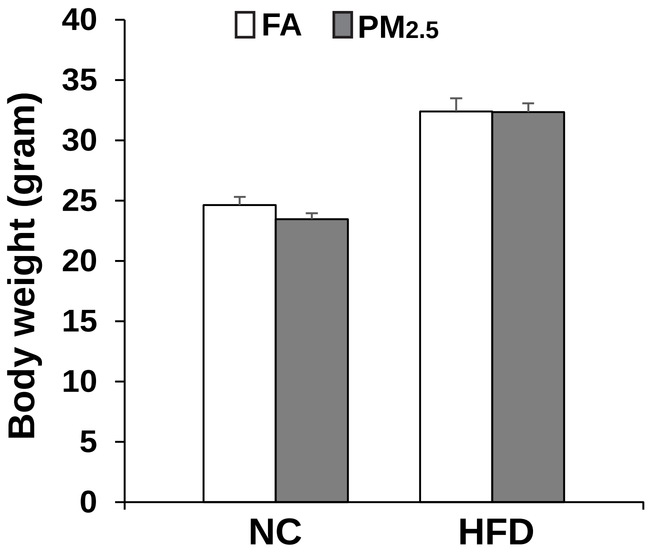

Supplemental figure 1

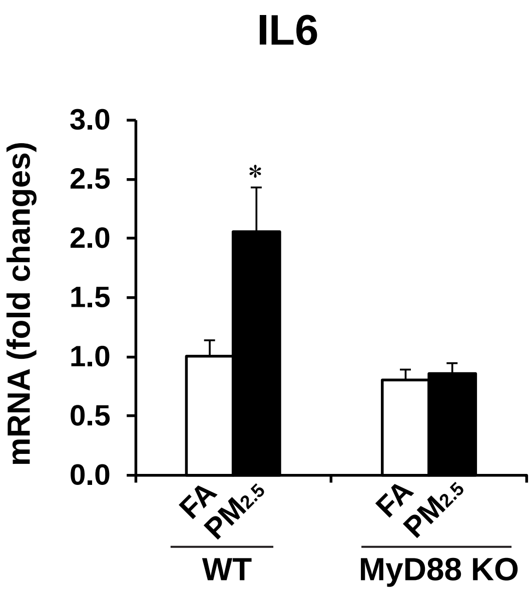

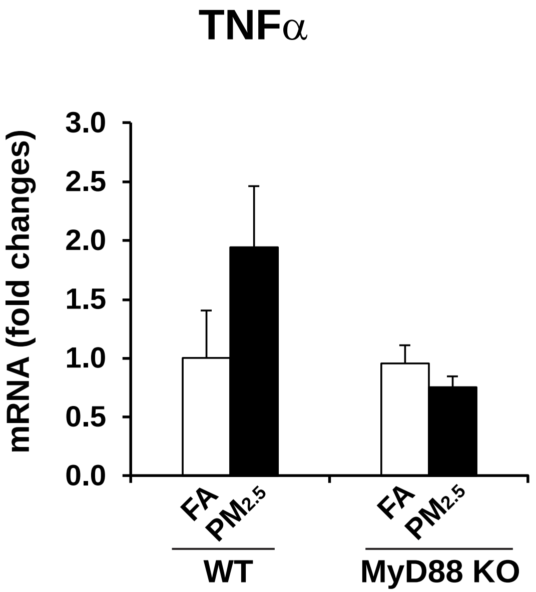

Supplemental figure 2

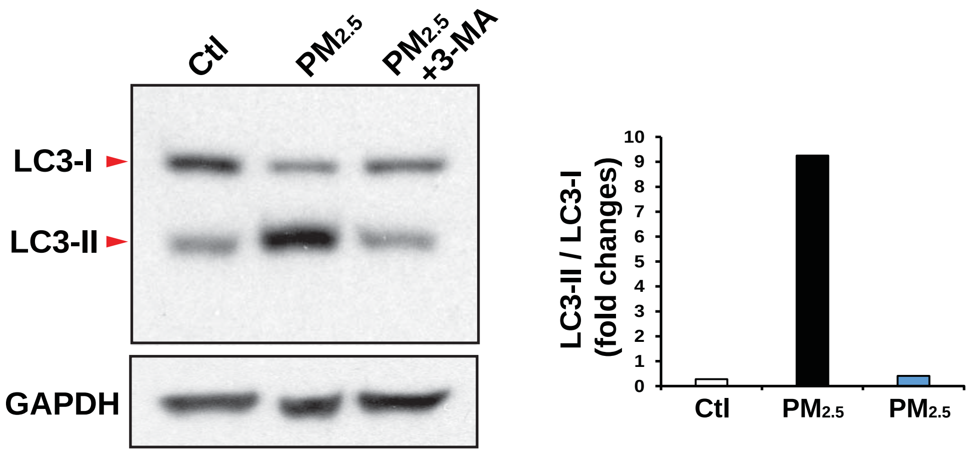

Supplemental figure 3

S-table 1

Sequence information for the quantitative real-time RT-PCR analysis in this study

Gene name	Forward primer	Reverse primer
Lc3b	GCT CTT TGT TGG TGT GTA	TCT TCT GTT GCT GTT GTC
Pdgf	TCA AGG TGG CCA AAG TGG AG	CTG GGT TCA GGT TGG AGG TC
Apoa4	GCA TCT AGC CCA GGA AAC TG	ATG TAT GGG GTC AGC TGG AG
Tfeb	CAG AAG CGA GAG CTA ACA GAT	TGT GAT TGT CTT TCT TCT GCC G
Ctsa	GCT ACC TCA GAG CAT CGG AC	GTT AAG CCA AAG CAC CAC GG
Gba	GCC TCC CAG AAG AAG ACA CC	ATA TCC CCT GGC TGA CCC TT
P62	GCT GAA GGA AGC TGC CCT AT	GCC TTC ATC CGA GAA ACC CA
Pparα	GGGAACTTAGAGGAGAGCCAAG	CCATGTTGGATGGATGTGGC
Sirt1	GCC GCG GAT AGG TCC ATA TAC T	GCC ACA GCG TCA TAT CAT CCA
Pparγ1	GGT GTG ATC TTA ACT GCC GGA	ACC TGA TGG CAT TGT GAG ACA
Fasn	GGAGGTGGTGATAGCCGGTAT	TGGGTAATCCATAGAGCCCAG
